# Intravenous Tissue Plasminogen Activator Can Be Safely Given without Complete Blood Count Results Back

**DOI:** 10.1371/journal.pone.0131234

**Published:** 2015-07-06

**Authors:** Yi Dong, Lumeng Yang, Jinma Ren, Deepak S. Nair, Sarah Parker, Jan L. Jahnel, Teresa G. Swanson-Devlin, Judith M. Beck, Maureen Mathews, Clayton J. McNeil, Yifeng Ling, Xin Cheng, Yuan Gao, Qiang Dong, David Z. Wang

**Affiliations:** 1 Department of Neurology, Huashan Hospital, State Key of Laboratory of Neurobiology, Fudan University at Shanghai, Shanghai, China; 2 Center for Outcomes Research, University of Illinois College of Medicine at Peoria, Peoria, IL, United States of America; 3 INI Stroke Network, OSF Healthcare System, Department of Neurology, University of Illinois College of Medicine at Peoria, Peoria, IL, United States of America; 4 Department of Neurology, The First Affiliated Hospital of Zhengzhou University at Henan, Zhengzhou, China; Massachusetts General Hospital, UNITED STATES

## Abstract

**Introduction:**

It is well known that the efficacy of intravenous (IV) tissue plasminogen activator (tPA) is time-dependent when used to treat patients with acute ischemic strokes.

**Aim:**

Our study examines the safety issue of giving IV tPA without complete blood count (CBC) resulted.

**Materials and Methods:**

This is a retrospective observational study by examining the database from Huashan Hospital in China and OSF/INI Comprehensive Stroke Center in United States. Patient data collected included demographics, occurrence of symptomatic intracranial hemorrhage, door to needle intervals, National Institute of Health Stroke Scale scores on admission, CBC results on admission and follow-up modified Rankin Scale scores. Linear regression and multivariable logistic regression analysis were used to identify factors that would have an impact on door-to-needle intervals.

**Results:**

Our study included120 patients from Huashan Hospital and 123 patients from INI. Among them, 36 in Huashan Hospital and 51in INI received IV tPA prior to their CBC resulted. Normal platelet count was found in 98.8% patients after tPA was given. One patient had thrombocytopenia but no hemorrhagic event. A significantly shorter door to needle interval (DTN) was found in the group without CBC resulted. There was also a difference in treatment interval between the two hospitals. Door to needle intervals had a strong correlation to onset to treatment intervals and NIHSS scores on admission.

**Conclusion:**

In patients presented with acute ischemic stroke, the risk of developing hemorrhagic event is low if IV tPA is given before CBC has resulted. The door to needle intervals can be significantly reduced.

## Introduction

For every minute of ischemia, 19 million neurons, 140 billion synapses and 7.5-mile long nerve fibers will die. To save neurons dying from ischemia, every second counts. Intravenous (IV) tissue plasminogen activator (tPA) remains the only effective IV treatment that can be given in any emergency room to patient with acute ischemic stroke (AIS). It has been well recognized that giving IV tPA early was associated with reduced mortality and symptomatic intracranial hemorrhage (sICH). Stroke patients received tPA have higher rates of functional independence at discharge and better chance of going home [1–4]. That is why reducing door to needle (DTN) interval when giving IV tPA is the key to resolve ischemia and save brain cells. The science supports the effort optimizing clinical process so that IV tPA could be given as soon as possible.

A recent large quality improvement study reported that among more than 30,000 patients treated with tPA, time of arrival after onset was inversely related to the DTN interval. For some unclear reasons, the earlier the patient arrived, the longer it took to get treated[5]. In clinical practice, many experienced stroke centers have difficulty achieving the guideline recommended 1-hour DTN time. Consequently many patients missed the opportunity to receive IV tPA. Given the strong association between the time of treatment and good outcome, many centers around the world have implemented strategies to shorten the DTN intervals. American Heart Association/American Stroke Association’s newest guideline recommends that the DTN interval be within 60 minutes[6]. German physicians have installed CT scanner in the ambulances[7]. The existing "code stroke" model at the Royal Melbourne Hospital was evaluated and restructured to include key components of the Helsinki model: 1) ambulance prenotification with patient details be alerted to the stroke team that meets the patient on arrival; 2) patients transferred directly from triage onto the CT table from the stretcher coming off the ambulance; and 3) tPA delivered in CT scanner area immediately after imaging and assessment[8].

It is well known that reviewing the eligibility criteria before considering IV tPA is time consuming. On the other hand, there is no evidence that harm has been done if these criteria are not strictly followed. A multicenter registery study showed that even in very old patients, the incidence of sICH did not increase after IV-tPA [9]. Another study concluded that there was no need to wait for coagulation results back before giving tPA [10]. Simplified Management of Acute Stroke Using Revised Treatment Criteria (SMART) trial only used the finding of acute hemorrhage as the absolute exclusion criteria [2]. All these studies suggested that simplifying the eligibility criteria had low risk of hemorrhage but could shorten the door to needle interval significantly.

### Aims

One of the eligibility criteria for IV TPA is to have the results of complete blood counts (CBC) back. If the platelet count is less than 100,000 per cubic millimeter, IV TPA should not be considered. This criterion was used during the initial National Institute of Neurological Disorders and Stroke (NINDS) trial for the purpose of avoiding hemorrhages since tPA had unknown risks for stroke patients at that time. To study if CBC really needs to be resulted before IV tPA, we studied the efficacy and safety in patients received the treatment with and without CBC back.

## Materials and Method

### Design and Patients

This is a retrospective observational study that included the patients treated with IV tPA in ultra-short time from Huashan Hospital (HS) in China and OSF/INI Comprehensive Stroke Center (INI) in the United States. Both institutions conducted quality improvement process to optimize the DTN interval. Protocols used by the two hospitals included 1) no labs required prior to initiation of IV tPA if clinically appropriate except blood glucose level; 2) stroke code alert to CT technologist; 3) CT read by treating neurologist. All patients were registered since 2009 and the time window of giving IV tPA has been extended to 4.5 hours. If a patient had a history of coagulopathy or was on any anticoagulant, then one must wait for CBC to result before IV tPA.

Since there was no evidence that harm had been done if these criteria were not followed strictly, HS implemented their protocol since Jun 2012 and INI began their efforts since Jan 2012. Patients who had CBC results first and then being given IV tPA were used as control group. All eligible patients received IV tPA without CBC back were included in this study.

Patient records/information was de-identified by C.M. prior to analysis. This study was approved by Peoria community IRB and Huashan Hospital IRB.

### Data collection

Both institutions used the European Cooperative Acute Stroke Study (ECASS) III inclusion and exclusion criteria [4,11] for administering IV tPA with the exception of not waiting for CBC results back. DNT was defined as the intervals from the time of arrival at the door of emergency room to the beginning of tPA bolus. Onset to treatment (OTT) interval was defined as the interval from the time patient last known normal to the beginning of tPA bolus. All demographic data including age, sex and race were collected.

Performed by stroke neurologists, stroke severity was measured by National Institute of Health Stroke Scale (NIHSS) scores on admission before tPA treatment. Complications such as sICH were recorded according to the findings of repeated CT scan or MRI 24hours after tPA treatment. CBC results were examined. Follow-up modified Rankin Scale (mRS) was done at the stroke clinic.

### Statistical analysis

Frequency/percentage and mean/standard deviation were reported for categorical and continuous variables, respectively. Multivariable linear regression analysis was used to examine the association between the DTN intervals (natural logarithms transformation) and factors related to tPA treatment. Previous studies and guidelines indicated 60 minutes of DTN was used as a cutoff point [6,11]. Multivariable logistic regression analysis was used to examine if tPA treatment before CBC resulted would have a significant impact on DTN intervals (over 60 minutes). Other controlled covariates included age, sex, sICH, admission NIHSS, mRS and hospitals. All statistical analyses were performed with the Statistical Analysis System (SAS) software. Statistical significance was taken at the two-tailed 0.05 level.

## Result

One hundred twenty patients in HS and 123 patients in INI met the inclusion criteria for our study. Among them, 36(30%) in HS and 51 (59%) in INI had IV TPA prior to their CBC resulted. Their average age was 68.6±12.2 years old and 105(43.2%) were female. Baseline clinical characteristics were listed in Table 1.

**Table 1:** Demographic and clinical characteristics of AIS patients treated with IV tPA.

Items	N(%)
Age (mean± SD)	68.6±12.2
> = 70 years old	127 (52.3)
<70 years old	116 (47.7)
Sex	
Male	139(56.8)
Female	105(43.2)
Race	
Caucasian	109(44.9)
African American	9(3.7)
Asian	120(49.4)
Other	5(2.1)
NIHSS at Admission (points, mean±std)	10.1±7.5
Door to needle (minute, mean±std)	64.0+39.1
Onset to treatment (minute, mean±std)	163.0+93.9
mRS (points, mean±std)	2.5±2.2
0	62(25.5)
1	45(18.5)
2	24(9.9)
3	34(14.0)
4	22(9.1)
5	20(8.2)
6	36(14.8)
sICH	
Yes	14(5.8)
No	229(94.2)
Hospital code	
INI	139(57.2)
HS	104(42.8)
Group	
Without CBC	85(35)
With CBC	158(65)

SD = standard deviation; NIHSS = National Institute of Health Stroke Scale; mRS = modified Rankin Scale; sICH = symptomatic intracranial hemorrhage; INI = Illinois Neurological institute/OSF Comprehensive stroke center; HS = Huashan Hospital; CBC = complete blood counts

Normal CBC results were found in 86 (98.8%) patients after tPA has been given. Only one patient had a platelet count of 88,000 but she did not experience any hemorrhagic event. Overall, 14 (5.8%) had sICH.

Our study showed that in the group without CBC results back, the DTN interval was reduced by an average of 28.3 minutes (relative reduction as 38.3%). As Table 2 depicted, DTN interval was associated with age (p = 0.012), hospitals (p<0.001) and CBC groups (p<0.001) in the univariable linear analysis. Similar results were seen in multivariable general linear model after adjustment for covariables (Table 2).

**Table 2:** Determinants of door to needle time (logarithms transformed) linear regression model.

Items	Unadjusted			Adjusted		
	Beta	Std Err	P value	Beta	Std Err	P value
Age (y.o)	-0.009	0.003	0.012	-0.002	0.003	0.354
Sex (ref: male)	-0.018	0.092	0.051	-0.005	0.078	0.954
Admission NIHSS (point)	-0.009	0.058	0.112	-0.116	0.051	0.025*
sICH (ref: yes)	0.008	0.197	0.966	-0.011	0.158	0.944
mRS (ref:mRS 2 or more)	0.073	0.092	0.431	-0.078	0.082	0.339
Hospital (ref: HS)	-0.828	0.008	<0.001	-0.762	0.079	<0.001*
CBC (ref: with CBC)	-0.529	0.090	<0.001	-0.291	0.008	0.004*

*All door-to-needle intervals and admission NIHSS were transformed as natural logarithms. The door to needle interval was a part of onset to treatment (OTT) time and the ethnicity of patients was distinctive from hospitals. We removed ethnicity and OTT time from this linear regression model. Std Err = standard error; NIHSS = National Institute of Health Stroke Scale; sICH = symptomatic intracranial hemorrhage; mRS = modified Rankin Scale were divided into two groups, favorable outcome as mRS scores 0 or 1, unfavorable outcome as mRS scores 2 or more; HS = Huashan Hospital; CBC = the complete blood counts result back before tPA

**Fig 1:**
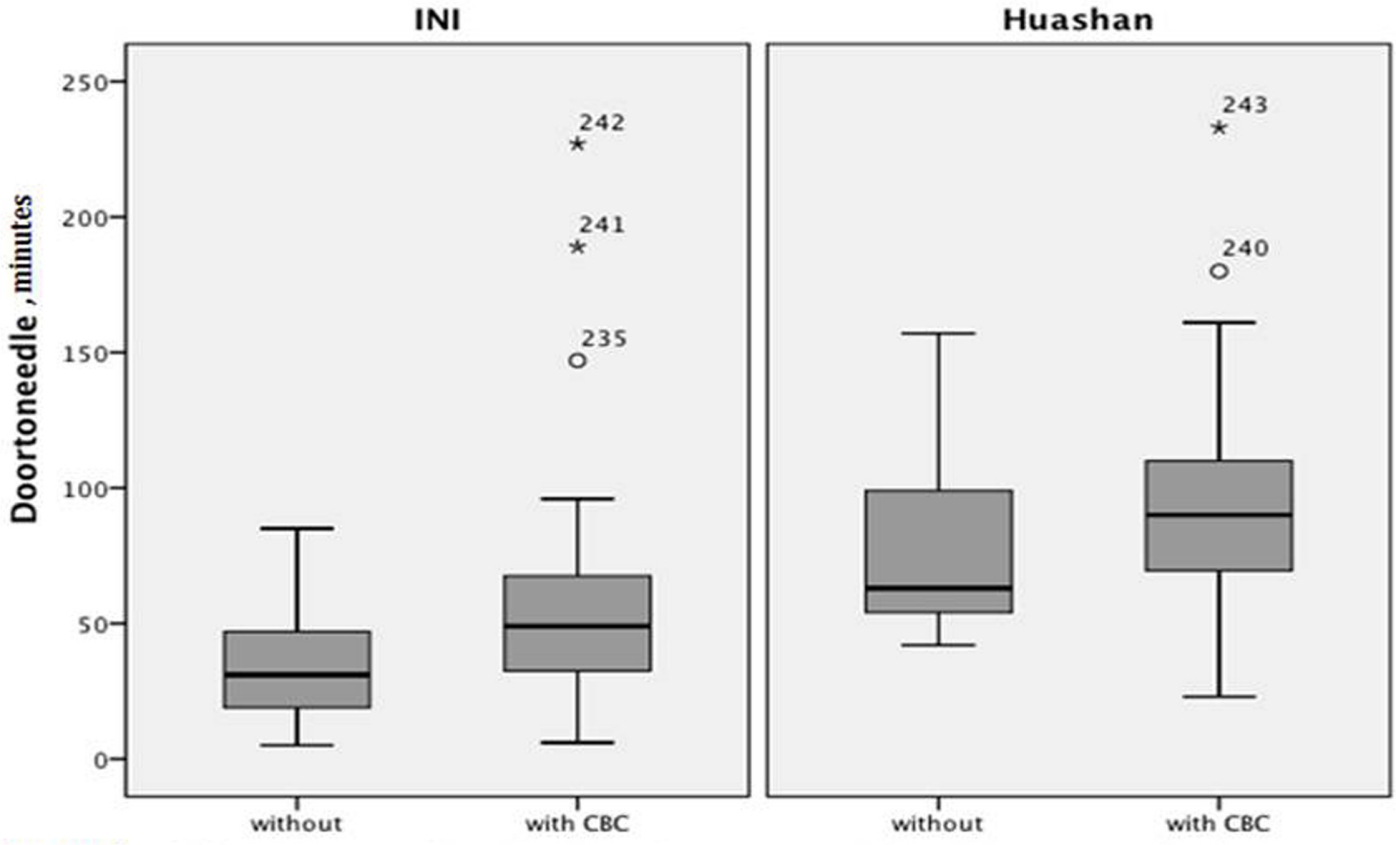
Different DTN distributions between with/without CBC before tPA. DTN = door to needle intervals, CBC = complete blood count, tPA = tissue plasminogen activator; INI = Illinois Neurological Institute/OSF Comprehensive stroke center.

In the logistic regression analysis, DTN interval within 60 minutes would be more likely reachable without a CBC result back (OR 0.25, 95%CI 0.12–0.50). Patients presented with milder symptoms (OR 1.72, 95%CI 1.10–2.68, p = 0.018) or treated at INI (OR 2.38, 95%CI 1.14–4.96) had shorter DTN intervals. Also, those who had been treated within the standard DTN interval (OR 1.22, 95%CI 1.02–1.46) had greater chance to achieve better outcome (mRS 0–1) (Fig 1 and Table 3).

**Table 3:** The Comparison on door to needle intervals more of > 60 minutes in logistic regression model analysis.

	Unadjusted			Adjusted		
	OR	95%CI	P	OR	95%CI	P
Age(y.o)	1.01	0.99–1.03	0.472	0.99	0.96–1.02	0.516
Sex(ref: male)	1.32	0.79–2.21	0.286	0.85	0.43–1.66	0.630
Admission NIHSS (point) (point)NIHSS	1.23	0.89–1.69	0.217	1.72	1.10–2.68	0.018*
sICH	0.67	0.22–2.05	0.481	0.48	0.12–2.01	0.317
mRS (ref:mRS 2 or more)	1.14	0.69–1.91	0.604	2.38	1.14–4.96	0.021*
Hospital (ref: HS)	10.45	5.75–18.99	<0.0011	12.21	6.08–24.52	<0.001* *
CBC(ref: with CBC)	5.06	2.75–9.29	<0.0011	3.78	1.87–7.66	0.002*

According to the door to needle (DTN) intervals of > 60 minutes or not, the patients were divided into two groups and related factors were assessed. Since DTN is a more accurate measure and the onset to treatment (OTT) intervals along with the ethnicity of patients were quite different from these two hospitals, we removed race and OTT from this analysis. OR = odds ratio; CI = confidential interval; NIHSS = National Institute of Health Stroke Scale; sICH = symptomatic intracranial hemorrhage; mRS = modified Rankin Scale were divided into two groups, favorable outcome as mRS scores 0 or 1, unfavorable outcome as mRS scores 2 or more; HS = Huashan Hospital; CBC = the complete blood counts result back before tPA.

## Discussion

Every second counts when it comes to save brain cells from ischemic stroke. IV tPA is still the only Food and Drug Administration approved IV treatment for acute ischemic stroke. However, many precious minutes have been wasted when going over the list of complex inclusion/exclusion criteria before giving IV tPA. This practice has been adapted because it was used during the original NINDS tPA stroke trials [5]. It is unlikely that IV tPA may cause more sICH when these criteria are not followed strictly.

Recent studies have shown that simplifying the criteria still brought about good outcome [2,8,12]. Many studies have confirmed that age and PT/APTT value [9,10] are not essential in patients who are not on any anticoagulants. By doing so, they shortened DTN interval by at least 20 minutes. Patients in SMART trial showed better outcome and less sICH than those in ECASS III [2]. Our study showed that the chance of having an abnormal CBC was rare and abnormal platelet count may not produce adverse outcome.

There are differences in healthcare environment and protocols between the two institutions. In China, tPA protocol was still being developed at both physician and patient level and written consent must be obtained. In United States, emergency service system (EMS) pre-notified the stroke team that would meet the patient on arrival at the door, which shortened the DTN interval significantly in the US. Despite such differences in practice, our studies still showed a 38.3% reduction of DTN interval by not waiting for the results of CBC before giving IV tPA at both institutions. At HS, they were able to achieve the goal of 60 minutes DTN interval by not waiting for CBC results, which helped to offset the time needed to obtain the written consent.

Our study had limitations. First, it was a retrospective study. Not all data elements were predefined. Second, not all data elements were matched at the two institutions which may contribute to sampling error and data inhomogeneity. Third, there could be a patient selection bias. Patients with large strokes were easier to be recognized and therefore treated than those with rapid improvement or minor stroke. More case-control studies should be designed to re-evaluate the criteria of tPA treatment.

## Conclusion

In patients presented with AIS who are candidates for IV tPA, the risk of hemorrhage is low if IV tPA is given first before CBC result is available. Such practice significantly reduced the DTN interval, which can in turn save more brain cells from dying of ischemia. Our study confirmed that the shorter the DTN interval, the better the outcome for the stroke patient. In addition the rate of sICH is low[13]. Therefore, having CBC results back is not essential when IV TPA is considered. Our study also proved that quality practice improvement can be effectively implemented worldwide.
